# Nrf2-driven TERT regulates pentose phosphate pathway in glioblastoma

**DOI:** 10.1038/cddis.2016.117

**Published:** 2016-05-05

**Authors:** F Ahmad, D Dixit, V Sharma, A Kumar, S D Joshi, C Sarkar, E Sen

**Affiliations:** 1 National Brain Research Centre, Department of Molecular and Cellular Neuroscience Division, Manesar, Gurgaon, Haryana 122051, India; 2 All India Institute of Medical Sciences, Department of Pathology, Ansari Nagar East, New Delhi 110029, India

## Abstract

Given the involvement of telomerase activation and dysregulated metabolism in glioma progression, the connection between these two critical players was investigated. Pharmacological inhibition of human Telomerase reverse transcriptase (hTERT) by Costunolide induced glioma cell apoptosis in a reactive oxygen species (ROS)-dependent manner. Costunolide induced an ROS-dependent increase in p53 abrogated telomerase activity. Costunolide decreased Nrf2 level; and ectopic Nrf2 expression decreased Costunolide-induced ROS generation. While TERT knock-down abrogated Nrf2 levels, overexpression of Nrf2 increased TERT expression. Inhibition of hTERT either by Costunolide, or by siRNA or dominant-negative hTERT (DN-hTERT) abrogated (i) expression of Glucose-6-phosphate dehydrogenase (G6PD) and Transketolase (TKT) – two major nodes in the pentose phosphate (PPP) pathway; and (ii) phosphorylation of glycogen synthase (GS). hTERT knock-down decreased TKT activity and increased glycogen accumulation. Interestingly, siRNA-mediated knock-down of TKT elevated glycogen accumulation. Coherent with the *in vitro* findings, Costunolide reduced tumor burden in heterotypic xenograft glioma mouse model. Costunolide-treated tumors exhibited diminished TKT activity, heightened glycogen accumulation, and increased senescence. Importantly, glioblastoma multiforme (GBM) patient tumors bearing TERT promoter mutations (C228T and C250T) known to be associated with increased telomerase activity; exhibited elevated Nrf2 and TKT expression and decreased glycogen accumulation. Taken together, our findings highlight the previously unknown (i) role of telomerase in the regulation of PPP and glycogen accumulation and (ii) the involvement of Nrf2-TERT loop in maintaining oxidative defense responses in glioma cells.

Telomerase is a ribonucleoprotein, composed of human telomerase reverse transcriptase (hTERT), human telomerase RNA subunit (TERC) and the telomerase-associated protein (TEP1). Telomere maintenance is required for long-term cellular growth and survival,^1^ and high telomerase levels are observed in most human malignancies.^2^ hTERT – the catalytic subunit of human telomerase – has a key role in the control of telomerase activity;^3^ and hTERT inhibition induces cancer cell apoptosis and limits tumor growth.^4, 5^ hTERT overexpression affects mitochondrial function and survival responses in cancer cells by regulating reactive oxygen species (ROS) production,^6^ and chemotherapeutic agents with ROS enhancing ability effectively kills cancer cells through elevation of oxidative stress.^7^

TERT promoter mutations that lead to enhanced expression of telomerase occur in several cancers including glioma.^8^ The prevalence of TERT promoter mutations is remarkably high in adult glioblastoma multiforme (GBM).^9^ Telomerase protects mitochondrial function under oxidative stress,^10^ and mitochondrial localization of hTERT induces apoptosis after oxidative stress.^11^ Also, the canonical telomeric protein TIN2 is post-translationally processed in mitochondria and regulates mitochondrial oxidative phosphorylation.^12^ Telomerase dysfunction represses mitochondrial function through p53; and this mitochondrial dysfunction is concomitant with compromised OXPHOS, decreased ATP generation, and increased oxidative stress.^13^ These findings along with the observation that anti-telomerase therapy triggers mitochondrial adaptive mechanisms in cancer^14^ clearly indicate that cross-talk between telomeric proteins and mitochondria is linked with metabolic reprograming.

p53 has a crucial role in the cellular response to telomere dysfunction,^15^ and p53-induced metabolic modeler TIGAR affects glycolysis and pentose phosphate pathway (PPP).^16^ The PPP generates ribose 5-phosphate (R5P) which has a crucial role in nucleotide synthesis, and NADPH as reducing equivalents. Besides, activation of ROS-p53/Nrf2 signaling pathway is known to induce apoptosis.^17^ Oxidative stress-mediated activation of transcription factor Nrf2 induces expression of protective antioxidant genes.^18^ Nrf2-mediated regulation of PPP affects glucose metabolism and ROS homeostasis in cancer cells.^19^ By promoting metabolic activities, Nrf2 supports cell proliferation and contributes to cancer development.^20^ Telomere dysfunction is also associated with senescence,^21^ and glycogenesis has been linked to cellular senescence.^22^ Importantly, glycogen metabolism is one among the different metabolic adaptation strategies undertaken by cancer cell for its survival. As telomerase inhibition is considered as exciting therapeutic possibilities for the treatment of human cancers,^23^ we investigated whether telomerase inhibitor Costunolide^24^ could affect survival of glioma cells through regulation of its metabolic program.

## Results

### ROS-dependent p53 regulates telomerase activity in glioma cells

**Figure 1:**
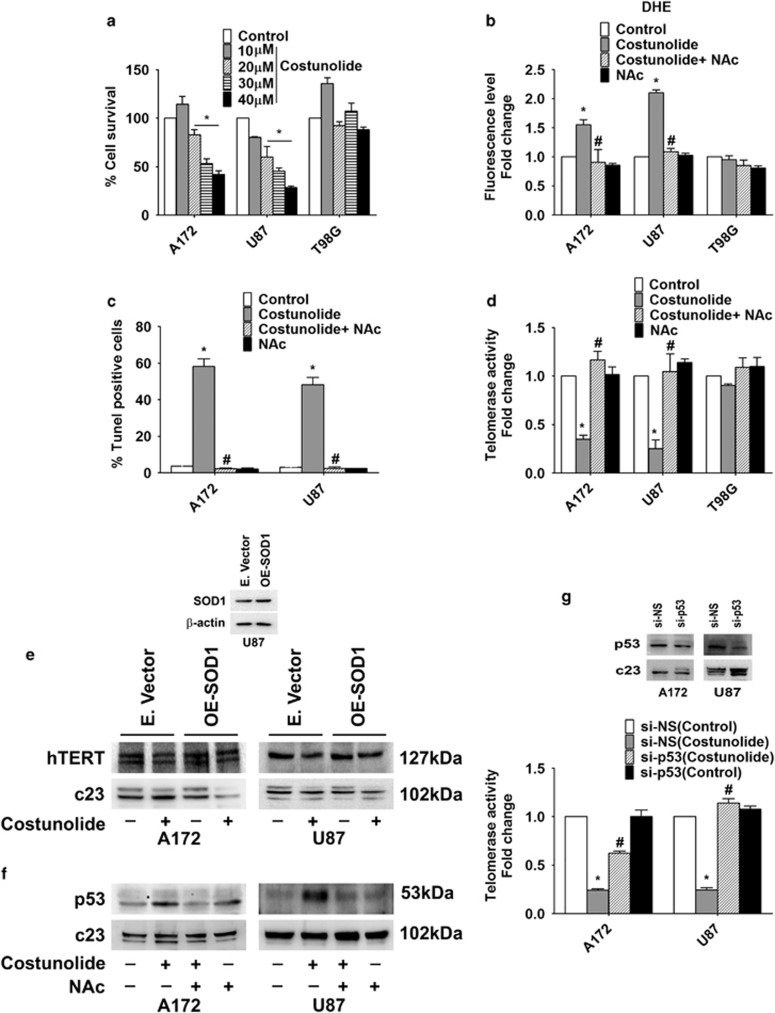
Costunolide induces glioma cell death and decreases telomerase activity in ROS/p53-dependent manner. (**a**) Costunolide reduces viability of A172 and U87MG glioma cells, but has no effect on p53 mutant T98G cells. Graph shows percent change in viability upon treatment with different doses of Costunolide for 24 h as determined by MTS assay. (**b**) The increase in DHE fluorescence induced upon Costunolide treatment is abrogated in the presence of ROS inhibitor NAc. (**c**) Costunolide-induced increase in TUNEL-positive cells is abrogated in the presence of NAc. Graph depicts the percent of TUNEL-positive cells treated with Costunolide, NAc or both. (**d**) ROS inhibition rescues Costunolide-mediated decrease in telomerase activity. Graph shows fold change in telomerse activity over control in glioma cells treated with different combinations of Costunolide and NAc. (**e**) Overexpression of SOD-1 rescues Costunolide-mediated decrease in hTERT expression. Following transfection of glioma cells with SOD-1 overexpression construct for 48 h, cells were treated in the presence and absence of Costunolide for an additional 24 h and hTERT levels were determined. Inset demonstrates SOD-1 expression in transfected cells. (**f**) Costunolide-induced increase in p53 expression is ROS dependent. Western blot images depicting p53 levels in cells treated with Costunolide in the presence or absence of NAc. (**g**) siRNA-mediated knock-down of p53 prevents Costunolide-mediated decrease in telomerase activity. Graph represents the telomerase activity of glioma cells transfected with either p53 siRNA or scrambled (NS) siRNA and treated with Costunolide. Values are expressed as fold change over control. Inset confirming the transfection efficiency of p53 siRNA. Values in the graph (**a**–**d** and **g**) represent the means±S.E.M. from three independent experiments. *Significant change from control, ^#^Significant change from Costunolide-treated cells (*P*<0.05). Blots (**e** and **f**) are representative images of three independent experiments showing similar results. Blots were re-probed for c23 to establish equivalent loading

Costunolide induced glioma cell death in a dose-dependent manner (Figure 1a). Although Costunolide induced death in A172 and U87MG glioma cells, it had no effect on the viability of p53 mutant glioma cell T98G (Figure 1a). Given the importance of p53 in cellular response to telomere dysfunction, the unresponsive of p53 mutant T98G cells to Costunolide can be explained. As ~50% decrease in cell viability was observed in A172 and U87MG glioma cells upon treatment with a 30-*μ*M concentration of Costunolide for 24 h (Figure 1a), treatment with 30 *μ*M dose of Costunolide for 24 h was used to dissect its mechanisms of action in subsequent experiments. As elevation of basal ROS level triggers glioma cell apoptosis,^25^ and as Costunolide induced ROS triggers apoptosis,^26^ the levels of ROS in Costunolide-treated cells were determined. Although Costunolide induced ROS production in glioma cell lines A172 and U87MG bearing WT p53, no increase in ROS was observed in p53 mutant T98G (Figure 1b). The ability of ROS inhibitor NAc to abrogate Costunolide induced TUNEL-positive cells (Figure 1c) indicated that Costunolide-mediated glioma cell death is ROS dependent. As we have previously reported ROS-dependent telomerase activity in glioma cells,^27^ we investigated relationship between the two in Costunolide-treated cells. The decrease in telomerase activity observed upon Costunolide treatment was rescued in the presence of ROS inhibitor (Figure 1d). No such decrease in telomerase activity was observed in Costunolide-treated T98G cells (Figure 1d). We have previously reported that knock-down of SOD-1 which is crucial in maintaining cellular redox homeostasis increases sensitivity of glioma cells to chemotherapeutics-induced apoptosis through elevation of ROS levels.^28^ As SOD-1 acts as a major defense against ROS, we investigated whether overexpression of SOD-1 could reverse the effect of Costunolide-mediated changes in TERT expression. The ability of Costunolide to decrease TERT expression was rescued upon SOD-1 overexpression. This further confirmed the involvement of oxidative stress in the regulation of TERT expression (Figure 1e). As p53 is activated upon telomere dysfunction,^15^ the status of p53 in Costunolide-treated cells was investigated. Costunolide-induced increase in p53 levels was abrogated upon NAc treatment (Figure 1f). However, Costunolide had no effect on p53 expression in p53 mutant T98G cells (data not shown). Moreover, Costunolide-induced increase in ROS levels remained unaffected upon co-treatment with p53 inhibitor Pifithrin α (data not shown), suggesting that although ROS regulates p53 levels in Costunolide-treated cells that latter has no effect in regulating ROS production.

Similar to results obtained upon pharmacological inhibition of hTERT, siRNA-mediated hTERT inhibition induced p53 levels (Supplementary Figure 1A). Costunolide induced Caspase-3 and 8 activity (Supplementary Figure 1B and C), elevated Bax and Cytochrome c, and decreased Bcl2 levels in an ROS-dependent manner (Supplementary Figure 1D). As repression of hTERT by p53 is known,^29^ the role of p53 in regulating telomerase activity in Costunolide-treated cells was investigated. siRNA-mediated knock-down of p53 rescued Costunolide-mediated decrease in telomerase activity to a significant extent (Figure 1g).

### Existence of Nrf2-TERT loop in glioma cells

**Figure 2:**
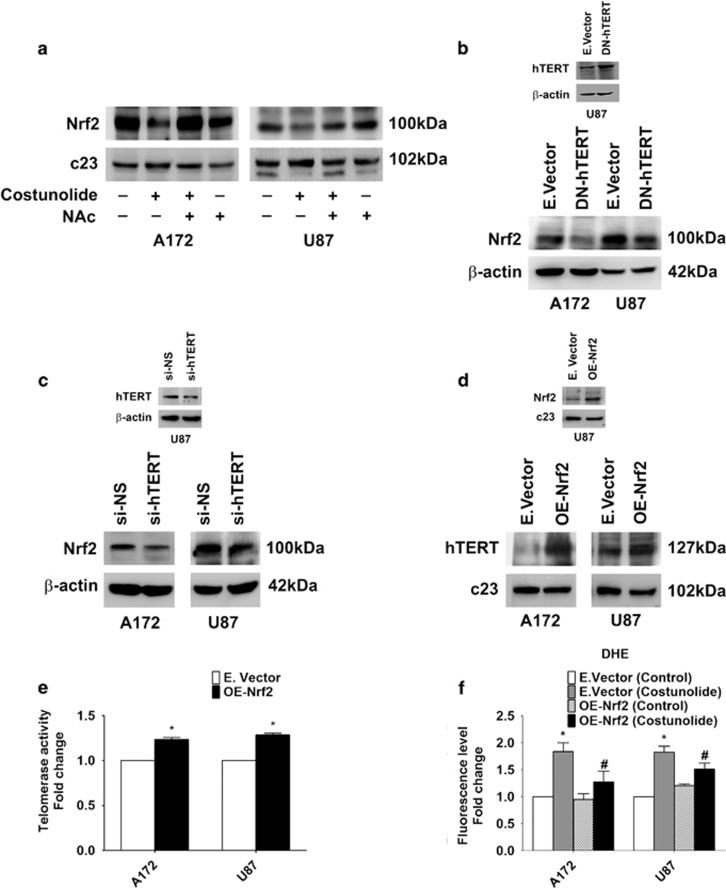
Existence of Nrf2-TERT regulatory loop in glioma cells. (**a**) Costunolide decreases Nrf2 levels in an ROS-dependent manner. Western blots showing Nrf2 levels in nuclear extracts of glioma cells treated with Costunolide in the presence and absence of NAc. (**b**) Transfection with DN-hTERT construct decreases Nrf2 levels in glioma cells, as demonstrated by western blot analysis. Inset confirming hTERT levels upon with transfection with DN-hTERT construct. (**c**) Western blot analysis demonstrating decreased Nrf2 levels in cells upon siRNA-mediated knock-down of hTERT. Inset confirming the transfection efficiency of hTERT siRNA. (**d**) Nrf2 overexpression increases TERT expression and (**e**) telomerase activity. Inset in (**d**) demonstrates Nrf2 expression in transfected cells. (**f**) Costunolide-induced ROS generation is abrogated upon Nrf2 overexpression. The graph represents DHE fluorescence intensity in cells transfected with either Nrf2 overexpression construct (OE-Nrf2) or empty vector and treated in the presence or absence of Costunolide. Fluorescence intensity values are expressed as fold change over control. Blots (**a**–**d**) are representative images of three independent experiments showing similar results. Blots were re-probed for *β*-actin or c23 to establish equivalent loading. Values in (**e** and **f**) represent the means±S.E.M. of three independent experiments. *Denotes significant change from control or mock-transfected group, ^#^depicts significant change from Costunolide-treated cells (*P*<0.05)

As activation of ROS-p53/Nrf2 signaling pathway is known to induce apoptosis,^17^ the status of Nrf2 in Costunolide-treated cells with heightened ROS and p53 level was investigated. A decrease in Nrf2 levels was observed in Costunolide-treated cells in an ROS-dependent manner (Figure 2a). However, no change in Nrf2 levels was observed in p53 mutant cell line T98G (Supplementary Figure 2A). A similar decrease in Nrf2 was observed in cells transfected with dominant-negative hTERT (Figure 2b) or hTERT siRNA (Figure 2c). This decrease in Nrf2 is crucial in regulation of hTERT, as overexpression of Nrf2 increased hTERT levels (Figure 2d). In addition, Nrf2 overexpression increased telomerase activity (Figure 2e) and decreased Costunolide-induced increase in ROS generation (Figure 2f).

### hTERT regulates PPP

**Figure 3:**
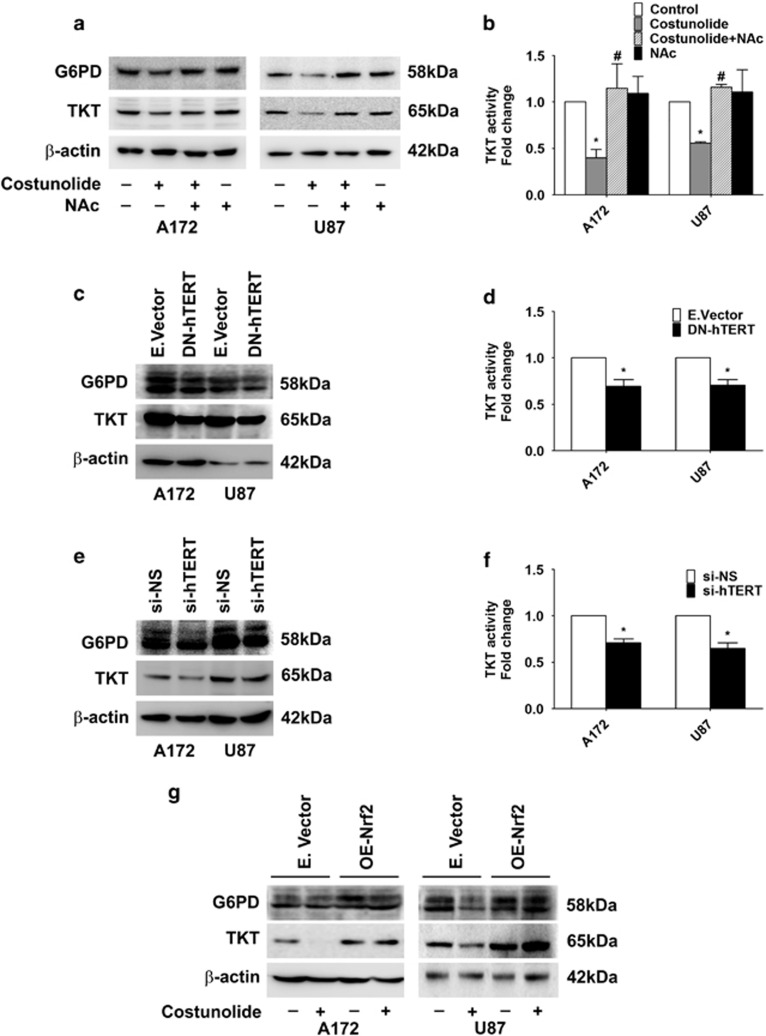
TERT regulates pentose phosphate pathway in glioma cells. (**a**) Costunolide decreases expression of G6PD and TKT in an ROS-dependent manner. Western blot demonstrating G6PD and TKT levels in cells treated with different combinations of Costunolide and NAc. (**b**) Costunolide-mediated decrease in TKT activity is rescued upon ROS inhibition. Graph showing TKT activity in cells treated with Costunolide in the presence and absence of NAc. (**c**) Transfection of glioma cells with DN-hTERT decreases G6PD and TKT levels in glioma cells, as depicted by western blot analysis. (**d**) Decreased TKT activity in glioma cells transfected with DN-hTERT construct. Values represent fold change in TKT activity in DN-hTERT-transfected cells over mock-transfected control. (**e**) Western blot demonstrating decreased G6PD and TKT levels upon siRNA-mediated knock-down of hTERT. (**f**) siRNA-mediated knock-down of TERT decreases TKT activity in glioma cells. Values represent fold change in TKT activity in TERT siRNA-transfected cells over NS-siRNA-transfected control. (**g**) Western blot analysis depicting G6PD and TKT levels in glioma cells transfected with either Nrf2 overexpression construct (OE-Nrf2) or empty vector and treated in the presence or absence of Costunolide. Blots shown in (**a**, **c**, **e**, and **g**) are representative images of three independent experiments showing an identical trend. Blots were re-probed for *β*-actin to establish equivalent loading. Values in (**b**, **d**, and **f**) are means±S.E.M. of three independent experiments. *Denotes significant change control or mock-transfected group, ^#^depicts significant change from Costunolide-treated cells (*P*<0.05)

PPP is crucial for glucose metabolic pathway associated with biosynthesis and antioxidant defense; and a deregulated PPP is known to promote oncogenesis.^30^ p53 regulates PPP by binding to glucose-6-phosphate dehydrogenase (G6PD)- the first and rate-limiting enzyme of the PPP.^31^ G6PD not only provides pentose sugars for RNA and DNA synthesis, but generates NAD(P)H that maintains pool of reduced glutathione to balance redox state.^32^ Moreover, Nrf2 regulates PPP and ROS homeostasis in cancer cells.^19^ As Nrf2 activates genes involved in the PPP,^20^ the status of G6PD and TKT in Costunolide-treated cells with diminished Nrf2 levels was investigated. Costunolide decreased G6PD and transketolase (TKT) expression, and this decrease was ROS dependent (Figure 3a). Costunolide-induced decrease in TKT level was concomitant with downregulation of TKT activity (Figure 3b), and treatment with NAc rescued Costunolide-induced decrease in TKT activity (Figure 3b). To confirm the direct involvement of hTERT in regulation of PPP, the expression of G6PD and TKT was determined in cells transfected with either DN-TERT or TERT siRNA. Decreased expression of both G6PD and TKT was observed in glioma cells upon transfection with TERT dominant-negative construct (Figure 3c) or siRNA-mediated knock-down of TERT (Figure 3e). This decrease in TKT level was accompanied by diminished TKT activity in cells transfected with either DN-TERT (Figure 3d) or TERT siRNA (Figure 3f). This indicated that a positive co-relation exists between hTERT and TKT in glioma cells. On analyzing the status of genes associated with glucose metabolism in Costunolide-treated cells using quantitative reverse transcriptase-PCR (QRT-PCR) based metabolism gene array, the transcript levels of G6PD and TKT – two important regulatory nodes in PPP were observed to be downregulated by greater than threefold (Supplementary Table 1).

### Nrf2 regulates TKT and G6PD expression

Nrf2 regulates PPP and ROS homeostasis in cancer cells,^19^ and Nrf2 activates genes involved in the PPP.^20^ Given that TERT regulates TKT expression and activity, and Nrf2 regulates TERT; we investigated the co-relation between PPP and Nrf2. An increase in both G6PD and TKT levels was observed upon Nrf2 overexpression (Supplementary Figure 2B). Also, overexpression of Nrf2 rescued Costunolide-mediated decrease in G6PD and TKT levels (Figure 3g), though the extent of rescue was cell line dependent.

### Costunolide increases senescence and glycogen accumulation

**Figure 4:**
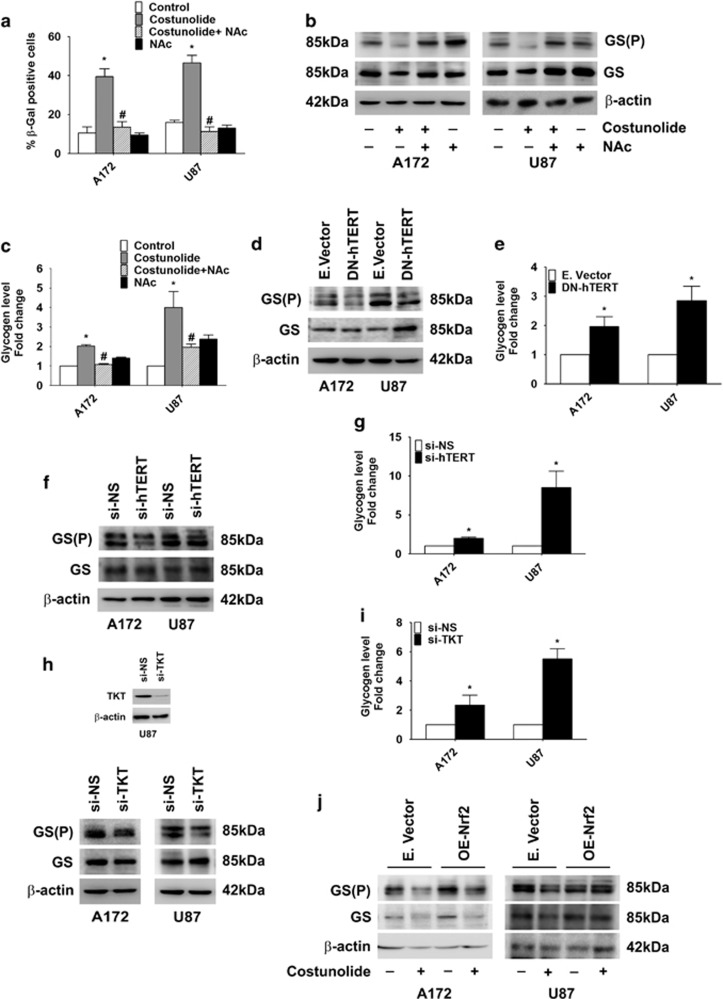
TERT regulates glycogen accumulation and senescence. (**a**) Costunolide induces glioma cell senescence in an ROS-dependent manner. The graph represents percentage *β*-gal-positive cells observed upon treatment with Costunolide in the presence and absence of NAc. (**b**) Costunolide decreases phospho-glycogen synthase GS(P) levels in an ROS-dependent manner. Western blot depicting GS(P) and GS levels in cells treated with different combinations of Costunolide and NAc. (**c**) Costunolide-mediated increase in glycogen accumulation is ROS dependent. Graph showing glycogen levels in cells treated with Costunolide in the presence and absence of NAc. (**d**) Transfection with DN-hTERT diminishes GS(P) levels and (**e**) increases glycogen accumulation in glioma cells. (**f**) siRNA-mediated knock-down of TERT decreases phosphorylated GS levels and (**g**) increases glycogen accumulation. (**h**) Western blot analysis demonstrating diminished GS(P) levels in glioma cells upon siRNA-mediated knock-down of TKT. (**i**) siRNA-mediated knock-down of TKT increases glycogen accumulation. (**j**) Western blot analysis depicting the effect of Nrf2 overexpression on Costunolide-induced changes in GS(P) and GS levels in glioma cells. Blots (**b**, **d**, **f**, **h**, and **j**) are representative of three independent experiments showing similar results. Blots were re-probed for *β*-actin to establish equivalent loading. The values in the graph (**a**, **c**, **e**, **g**, and **i**) represent the mean±S.E.M. from three independent experiments. *Significant change from control or non-specific siRNA or mock-transfected cells, ^#^significant change from Costunolide-treated cells (*P*<0.05)

Telomere dysfunction is associated with senescence,^21^ and inhibition of Nrf2 triggers stress-induced premature senescence.^33^ Costunolide-induced increase in *β*-gal-positive cells, indicative of senescence, was diminished in the presence of ROS inhibitor (Figure 4a). As glycogenesis has been linked to cellular senescence,^22^ the status of phosphorylated glycogen synthase GS(P) which is known to trigger glycogen accumulation^34^ was investigated. Costunolide-induced senescence was accompanied by decreased expression of GS(P) (Figure 4b) and increased glycogen accumulation (Figure 4c) in an ROS-dependent manner. The extent of glycogen accumulation was cell line dependent (Figure 4c).

### Glycogen accumulation in glioma cells is TERT driven

To confirm the involvement of hTERT in glycogen accumulation, GS(P) and GS levels were determined in cells transfected with either DN-TERT construct or TERT siRNA. A decrease in GS(P) expression (Figure 4d) concomitant with an increase in glycogen accumulation (Figure 4e) was observed upon transfection with DN-TERT. Similarly, decreased GS(P) level and increased glycogen accumulation observed upon siRNA-mediated knock-down of TERT indicated that glycogen metabolism in glioma cells is hTERT regulated (Figures 4f and g). As a positive correlation exists between hTERT and TKT, and as TERT was found to regulate glycogen accumulation; the role of TKT in glycogen accumulation was investigated. A decrease in GS(P) level (Figure 4h) and an increase in glycogen accumulation (Figure 4i) were observed upon transfection with TKT siRNA. As TERT regulates glycogen accumulation, and as Nrf2 regulates TERT; we next investigated the co-relation between Nrf2 and glycogen metabolism. An increase in GS(P) levels was observed upon Nrf2 overexpression (Supplementary Figure 2C). In addition, Nrf2 overexpression partially rescued Costunolide-mediated decrease in GS(P) levels with the extent of rescue being cell line dependent (Figure 4j).

### Costunolide impairs growth of tumor xenografts

**Figure 5:**
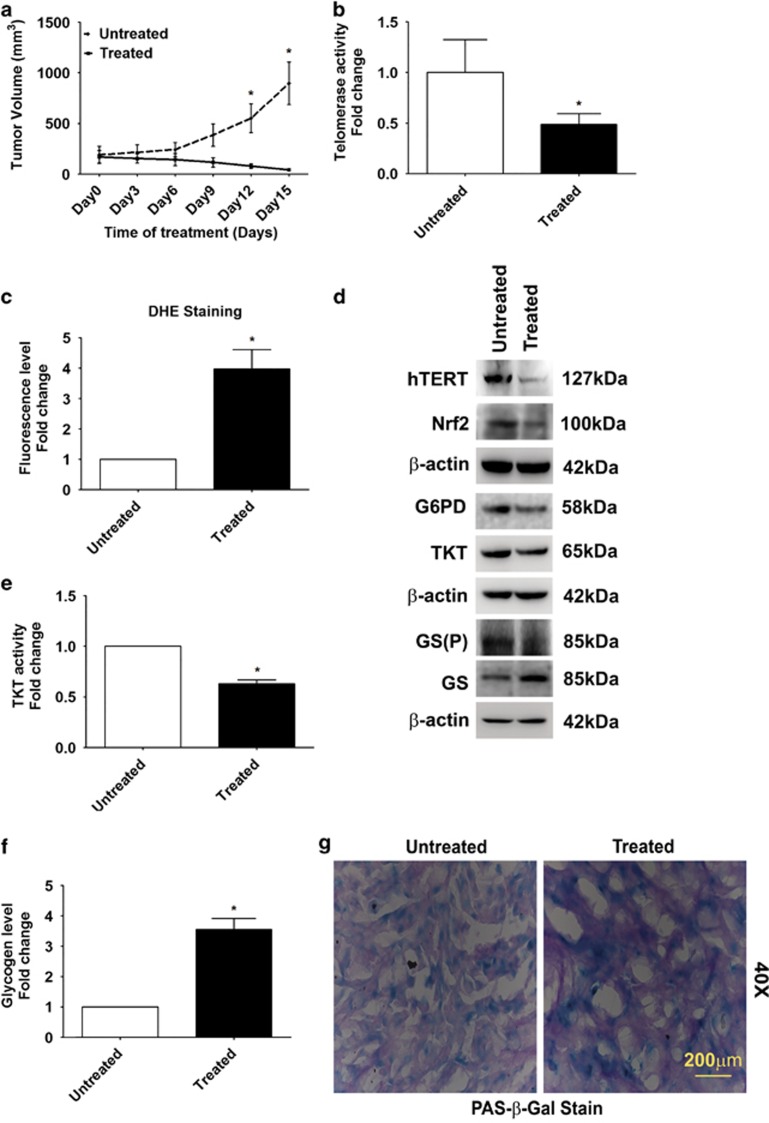
Costunolide inhibits pentose phosphate pathway and increases glycogen accumulation in heterotypic xenograft glioma model. (**a**) Significant reduction in tumor volume in Costunolide-treated glioma xenografts as compared with untreated groups (*n*=7). (**b**) Costunolide-treated tumors show decreased telomerase activity as compared with control groups (*n*=4). (**c**) Elevated ROS levels in Costunolide-treated tumors as compared with untreated group, as indicated by increased DHE fluorescence (*n*=4). (**d**) Western blot showing decreased hTERT, Nrf2, G6PD, TKT, and GS (P) levels in the total cell lysates prepared from Costunolide-treated tumors as compared with untreated groups. Blot is representative images (*n*=6). Blot was re-probed for *β*-actin to establish equivalent loading. (**e**) Decreased TKT activity and (**f**) elevated glycogen levels in Costunolide-treated tumors as compared with untreated controls. The values in graph (**b**, **c**, **e**, and **f**) indicate means±S.E.M. of *n*=4. Statistical analysis was done using unpaired Student's *t*-test. Results were considered as significant, when *P-*value was equal to or less than 0.05. *Denotes significant change from the untreated group. (**g**) Costunolide increases glycogen accumulation in cells undergoing senescence as demonstrated by increased PAS and *β*-glactosidase staining. Sections derived from Costunolide-treated U87 xenografts were co-stained for *β*-glactosidase and glycogen (PAS). Representative image from animals from each group is shown (*n*=4)

We next tested the ability of Costunolide to regulate tumor growth in glioma xenograft model by subcutaneously injecting cells into the flank of mice. The ability of Costunolide to significantly diminish tumor volume (Figure 5a) and weight (Supplementary Figure 3A) was accompanied by downregulation of telomerase activity (Figure 5b), increase in ROS level (Figure 5c), and elevated caspase 3/8 activity (Supplementary Figure 3B and C).

### Costunolide downregulates PPP and increases glycogen accumulation in glioma xenograft tumors

Costunolide-mediated decrease in tumor volume was accompanied by decreased expression of TERT, Nrf2, G6PD, TKT, and GS(P) levels (Figure 5d). Costunolide-treated tumors exhibited a decrease in TKT activity (Figure 5e) and an increase in glycogen accumulation (Figure 5f) as compared with untreated groups. An increase in senescence was also observed in tumors obtained from Costunolide-treated animals as compared with untreated controls (Supplementary Figure 3D). As senescence is associated with glycogen accumulation,^35^ the status of glycogen in senescent cells was investigated. An increased periodic acid Schiff (PAS) and *β*-galactosidase staining was observed in Costunolide-treated tumors as compared with untreated control (Figure 5g).

### TERT mutation in GBM is associated with increased TKT expression

**Figure 6:**
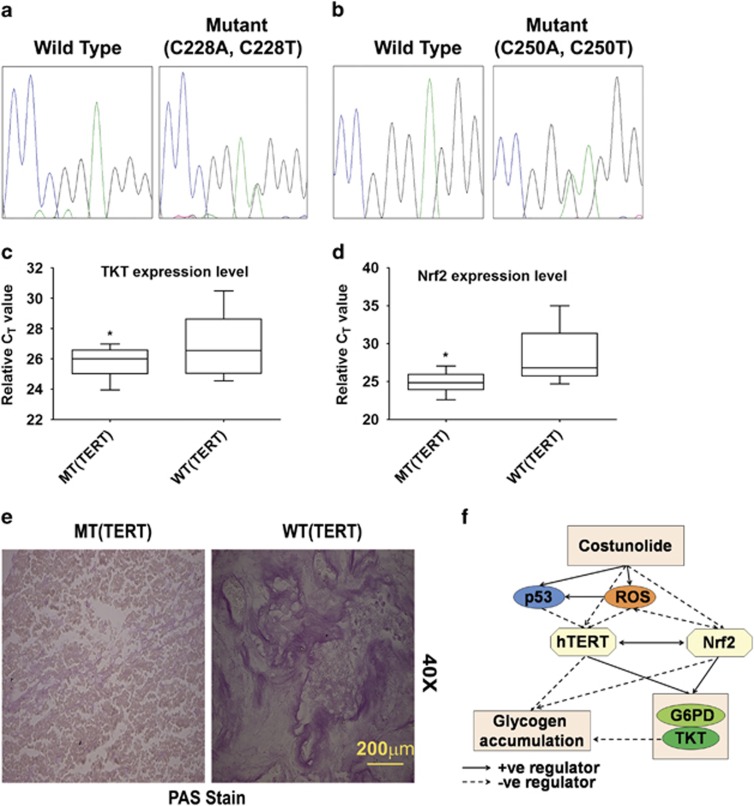
Co-relation between TERT, TKT, and Nrf2 in GBM tumors. (**a**) Sanger sequencing indicating C228T and (**b**) C250T mutation in GBM tumor samples. (**c**) qRT-PCR indicating elevated TKT and (**d**) Nrf2 mRNA expression levels in C228T- or C250T-mutated tumor samples. Result were analyzed using non-parametric Mann–Whitney test. (**e**) Immunohistochemistry showing decreased glycogen accumulation in GBM tumor bearing C228T or C250T mutation. MT and WT represent TERT mutant and wild-type GBM tumors, respectively. (**f**) Proposed model demonstrating the existence of Nrf2-TERT regulatory loop, and its involvement in regulating metabolic adaptation in glioma cells. *denotes significant change from WT

TERT promoter mutation occurs frequently in glioblastoma, with the C228T and C250T mutations accounting for majority of the alterations.^36^ Mutations at these sites are associated with increased telomerase activity. Sanger sequencing revealed TERT mutation in 12 out of 26 GBM patient tumors examined (C228T: 83% and C250T: 17%) (Figures 6a and b). Interestingly, except one, all the tumors bearing TERT mutations were p53 wild type (data not shown). As positive co-relation between telomerase activity and TKT was noted, the status of TKT in patients harboring TERT mutations at C228T and C250T was investigated. Real-time PCR analysis revealed an increase in TKT mRNA expression in tumors harboring these mutations (Figure 6c). A similar trend in Nrf2 mRNA expression was also observed in tumors bearing TERT mutations (Figure 6d). These changes were concomitant with decreased glycogen accumulation in TERT-mutated tumor samples, as compared with ones not harboring TERT mutation (Figure 6e). Taken together, these findings suggest that Nrf2-driven TERT has a crucial role in regulating PPP and glycogen accumulation in glioma cells (Figure 6f).

## Discussion

In addition to dysregulated metabolism, another notable feature that characterizes GBM is its obstinate resistance to current therapeutic regimen. Previous studies from our group have demonstrated the relationship between oxidative stress and resistance to apoptosis,^27, 28^ as well as metabolic adaptation in glioma.^25^ The prevalence of TERT promoter mutations in glioma has been suggested as potential biomarker;^9^ and oxidative stress-induced mitochondrial localization of hTERT is known to trigger apoptosis.^11^ As telomerase inhibition-mediated alternative lengthening of telomeres (ALT) rescues mitochondrial dysfunction and ROS, targeting ROS/mitochondria is regarded an efficient mechanism of enhancing anti-telomerase therapy.^14^ Also, we have shown ROS-dependent regulation of telomerase activity in glioma cells.^27^ To assess the involvement of telomerase in metabolic adaptation and oxidative defense mechanisms, we investigated the efficacy of telomerase inhibitor Costunolide with known ability to induce apoptosis in an ROS-dependent manner,^26^ as a potential anti-glioma target.

Nrf2 regulates ROS production by mitochondria and NADPH oxidase,^37^ and accumulation of Nrf2 in cancer cells offers protection against oxidative stress and chemotherapeutic agents.^38^ Besides, inhibition of Nrf2 enhances sensitivity to chemotherapeutics.^39^ It is possible that decreased Nrf2 level induced upon pharmacological inhibition or siRNA-mediated knock-down of TERT triggers ROS production to induce cell death. By increasing ROS accumulation while concomitantly disabling antioxidant defense mechanism through a decrease in Nrf2 levels, Costunolide triggers apoptosis by elevating oxidative stress. Though existing knowledge regarding TERT regulatory network have indicated the relevance of Nrf2 in affecting mitochondrial maintenance and oxidative defense mechanisms in tumors acquiring ALT upon telomerase extinction,^14^ our studies have highlighted the involvement of TERT-Nrf2 regulatory loop in the maintenance of redox homeostasis in glioma cells.

Our studies are in agreement with previous findings that increased glycogen accumulation following a decrease in glycogen phosphorylase induces ROS-dependent senescence, diminishes flux into PPP, and restricts glioma tumor growth.^22^ Moreover, increased senescence associated with glycogen phosphorylase depletion occurs via an ROS-dependent mechanism that leads to p53 activation.^22^ Interestingly, Nrf2 inhibition activates p53 and promotes premature senescence.^33^ Besides, Nrf2 is known to promote tumorigenesis through regulation of the PPP.^19^ It is possible that increased ROS levels contribute to p53-dependent induction of senescence in Costunolide-treated cells with depleted glycogen phosphorylase levels.^22^ Enhanced PPP glucose flux due to p53 inactivation not only accelerates glucose consumption but also directs glucose toward biosynthesis in tumor cells.^31^ Importantly, decreased glycogen breakdown induces cancer cell apoptosis by limiting glucose oxidation, as well as nucleic acid and *de novo* fatty acid synthesis.^40^ It is possible that Nrf2-TERT loop regulated PPP impairs glioma tumor growth by negatively affecting glycogen accumulation. Also, this ability of hTERT to drive cancer progression independent of its role in telomerase activity could explain high frequency of TERT reactivation in most human cancers.^41^ It is interesting to note that p53 responsive gene TIGAR provides nucleosides, NADPH, and anti-oxidants crucial for the development and promotion of tumor growth and proliferation.^42^ This ability of TIGAR to lower oxidative stress while promoting the PPP prevents ROS-mediated cell death while supporting anabolic pathways crucial for cell growth. It is tempting to speculate that the p53-TIGAR-PPP axis acts opposite to the p53-TERT-PPP axis, whereby the latter through induction of ROS triggers oxidative stress-induced apoptosis.

Interestingly, GBM patient tumors bearing TERT mutations that promote telomerase activation^9^ also exhibited elevated TKT and Nrf2 levels, and diminished glycogen accumulation. This observation in GBM patients further strengthen our findings that Nrf2-TERT regulatory loop promotes glioma progression by affecting cell survival, redox homeostasis, and metabolism. By underscoring the importance of hTERT as a driver of dysregulated metabolism and oxidative defence responses in glioma cells, this study paves way not only for a better understanding of aberrant metabolic programming in cancer but also for the design of effective therapeutics.

## Materials and Methods

### Cell culture and treatment

Human glioma cell lines A172, U87MG, and T98G obtained from American Type Culture Collection (ATCC, Manassas, VA, USA) and from ECACC (European Collection of Cell Cultures) were cultured in DMEM supplemented with 10% HI-FBS (Gibco, Grand Island, NY, USA) and penicillin (100 U/ml)/streptomycin (100 *μ*g/ml). On attaining semi-confluence, cells were switched to serum-free media (SFM) and after 4 h were pre-treated with ROS-specific inhibitor *N*-acetylcysteine (NAc; 2.5 mM, Sigma) for 2 h and subsequently co-treated in the presence or absence of Costunolide (30 *μ*M) (Tocris Bioscience, Bristol, UK) in SFM for 24 h. DMSO-treated cells served as controls.

### Transfection

Glioma cell lines were transiently transfected using Lipofectamine 2000 or Lipofectamine RNAiMax (Life Technologies-Invitrogen, Carlsbad, CA, USA) in Opti MEM according to the manufacturer's protocol, as described.^25^ Briefly, glioma cells were seeded onto 90/60 mm tissue culture dishes in DMEM, supplemented with HI-FBS, without antibiotics; and transfection with duplex (75 nmol/l) hTERT, (50 nmol/l) p53- or TKT-specific siRNAs, and non-specific (NS) siRNAs or with expression constructs for OE-SOD1, DN-hTERT, OE-Nrf2, pcDNA, and the corresponding empty vectors (E.Vector) was carried out using Lipofectamine RNAi Max reagent or Lipofectamine 2000 reagent, respectively as described.^25^ Cells transfected with NS or specific siRNA or expression vectors for 48 h were treated with Costunolide for additional 24 h, harvested and analyzed by western blot analysis. Control non-targeting (NS) siRNA as well as siRNAs targeting hTERT, p53, and TKT were obtained from Dharmacon (Thermo Fischer Scientific, Waltham, MA, USA). DN-hTERT construct (Plasmid #1775) and pcDNA Flag Nrf2 (Cat# 36971) were purchased from Addgene (Cambridge, MA, USA). pcDNA3.1 construct was purchased from Clontech (Mountain View, CA, USA). SOD-1 overexpression construct was a kind gift from NR Jana, NBRC.

### Determination of cell viability

Viability of cells treated with different dosages of Costunolide was assessed using the MTS assay (Promega, Madison, WI, USA) as described.^27^ Values were expressed as percentage change compared with controls.

### TUNEL assay

TUNEL assay was performed on glioma cells (10^4^) treated with Costunolide in the presence and absence of NAc as described previously,^28^ and percentage of cell death was determined from the number of TUNEL-positive cells (red) that colocalized with DAPI (blue) to the total number of cells taken from multiple fields.

### Measurement of ROS

Intracellular ROS generation in cells treated with Costunolide in the presence and absence of NAc was assessed by using fluorescent dye dihydroethidium (DHE) as described previously.^27^ ROS levels in tumor tissues obtained from control and Costunolide-treated glioma xenografts were measured as described previously.^25^

### TeloTAGGG telomerase PCR ELISA Plus

Telomerase activity was determined using the TeloTAGGG telomerase PCR ELISA Plus kit (Roche, Basel, Switzerland). Cells (2 × 10^5^) treated with Costunolide in the presence or absence of NAc or animal tissues were lysed according to the manufacturer's instruction. Equal amounts of protein (0.5 *μ*g) from cell and tissue extract were used. The assay was performed according to the manufacturer's protocol as described previously.^27^

### Western blot analysis

Western blot analysis was performed on protein lysates isolated from control or treated cells and tumor tissues as described previously^28^ using antibodies against Nrf2 (Abcam, Cambridge, UK), p53 (Cell Signaling, Danvers, MA, USA), and hTERT (Novus Biological, Cambridge Science Park, Cambridge, UK). Anti-phospho Glycogen Synthase (Ser641/Ser645) and glycogen synthase were obtained from Merck (Millipore, Billerica, MA, USA). G6PD and TKT were obtained from Sigma-Aldrich (St. Louis, MO, USA). Antibodies were purchased from Santa Cruz Biotechnology (Santa Cruz, CA, USA) unless otherwise mentioned. Secondary antibodies were purchased from Vector Laboratories Inc. (Burlingame, CA, USA). Blots were developed using chemiluminescent reagent (Millipore). After addition of chemiluminescent reagent, blots were exposed to Chemigenius Bioimaging System (Syngene, Frederick, MD, USA) for developing and images were captured using Genesnap software (Syngene). The blots were stripped and re-probed with HRP labeled anti-*β*-actin (Sigma) or C-23/Nucleolin to determine equivalent loading in whole cell or nuclear extract respectively, as described.^43^

### Assay for Caspase-3 and 8 activities

The Colorimetric Assay kits for Caspases-3 and 8 (Abcam) were used to determine the levels of active Caspases in cells treated with different combinations of Costunolide or NAc according to the manufacturer's instructions. Tissue lysates from untreated and Costunolide-treated tumor groups were also used to measure activity according to the manufacturer's instruction, as described previously.^25^

### TKT activity assay

TKT activity was measured using the TKT assay kit (Abcam) according to the manufacturer's instructions. Cell and tissue lysates were prepared from treated and untreated samples, equal protein samples were diluted in 50 *μ*l buffer and incubated for 2 h at 25 °C/300 r.p.m. in an incubator shaker. Plates were washed thrice with PBS and further incubated for 1 h with TKT antibody at 25 °C/300 r.p.m. Following incubation, the plate was washed and incubated with 50 *μ*l of HRP labeled antibody for 1 h at 25 °C/300 r.p.m. Plate was washed and 100 *μ*l of TMB development solution was added, reaction was stopped after 30 min by adding stop solution, and OD was recorded at 450 nm after every 10 min. TKT activity was calculated as described, and results were plotted as fold change over control.

### Glycogen accumulation assay

Glycogen level was measured using a Glycogen assay kit (BioVision Inc., Milpitas, CA, USA) according to the manufacturer's instructions. Equal amount of protein from control and treated cells, or tissue lysates of untreated and Costunolide-treated tumor groups were suspended in 50 *μ*l of lysis buffer. The lysates were further mixed with a reaction mixture (50 *μ*l/reaction), containing 46 *μ*l of development buffer, 2 *μ*l of development enzyme mix, and 2 *μ*l of OxiRed probe, and dispensed onto 96-well microplate for further incubation at 37 °C for 30 min. OD was measured at 570 nm from both samples and standard with the help of an ELISA plate reader (TECAN pro200, Männedorf, Zürich, Switzerland). Background correction was done by subtracting glucose readings from all glycogen readings obtained after 30 min. Readings were calculated as described and expressed as fold change over control.

### PAS staining for glycogen

Glycogen was detected in cells and tumor sections using PAS staining technique. Briefly, cells fixed with 4% paraformaldehyde for 15 min or fixed tumor sections rehydrated at room temperature were incubated in 1% periodic acid (Sigma-Aldrich) for 5 min, rinsed in water, and placed in Schiff's reagent (Sigma-Aldrich) for 15 min at room temperature. Finally, cells were washed with water. Amylase (Sigma-Aldrich) was used to verify that staining was specific for glycogen. Upon completion of reaction, slides were washed in running tap water for 5 min and left for air drying. Slides were mounted in DPX. Images were captured under Leica DMRXA2 bright field microscope (Wetzlar, Germany).

### *β*-Galactosidase staining

Cells treated with Costunolide either alone or in the presence of NAc were stained with the senescence-associated *β*-galactosidase staining solution (Sigma, St. Louis, MO, USA) as per the manufacturer's instructions, and percentage of SA-*β*-gal-positive cells calculated as described previously.^44^ *β*-Galactosidase staining was also performed on control and Costunolide-treated tumor samples.

### Human metabolism qRT-PCR array

qRT-PCR was performed using The Human Glucose Metabolism RT2 Profiler comprising 84 metabolism-related genes (SuperArray Biosciences, Hilden, Germany) as described previously.^25^ Five housekeeping genes were included on the array (B2M, HPRT1, RPL13A, GAPDH, and ACTB) to normalize the transcript levels. Results were analyzed as per user manual guidelines using integrated web-based software package for the PCR Array System (RT^2^ Profiler PCR Array Human Glucose Metabolism PAHS-006Z).

### Generation of heterotypic glioma xenografts

Heterotypic glioma xenografts were generated by injecting U87MG cells subcutaneously in the flank of anesthetized nude mice as described previously.^25^ After 15 days of injection, when measurable 5–6 mm tumors were formed, animals were divided randomly into 2 groups of 8 animals each and were administered either with vehicle or Costunolide (5 mg/kg body wt) intraperitoneally on alternate days for 20 days; following which animals were killed and tumor mass from each mouse was calculated and tissues were processed for subsequent experiments. Western blot analysis was performed on lysates prepared from control and Costunolide-treated tumor tissues as described previously.^25^ All the experimental procedures were in accordance with the guidelines of the Institutional animal ethics committee.

### qRT-PCR analysis

qRT-PCR analysis for TKT and Nrf2 expression was performed in MT (TERT Mutant) and WT (TERT Wild) confirmed GBM samples. Total RNA from pathologically confirmed samples was isolated; and cDNA was synthesized from 1 μg of total RNA by using a high-capacity cDNA reverse transcription kit (Applied Biosystems, Waltham, MA, USA). The real-time quantification of TKT and Nrf2 mRNA was performed with Power SYBR Green PCR master mix (Applied Biosystems) using a ViiA7 real time thermocycler (Applied Biosystems Inc.) for 40 cycles. All reactions were performed in duplicates and normalized with 18S rRNA as an internal control.

The primers used for RT-PCR are listed as follows:

TKT: Forward 5′-CCAAGTGATGGCGTTGCTACAG-3′

  Reverse 5′-TTGTCCGACCTGGAAGTCCTCA -3′

Nrf2: Forward 5′-CACATCCAGTCAGAAACCAGTGG-3′

  Reverse 5′-GGAATGTCTGCGCCAAAAGCTG-3′

18S: Forward 5′-GAGGGAGCCTGAGAAAACGG-3′

  Reverse 5′-GTCGGGAGTGGGTAATTTGC-3′

### TERT promoter mutation and TP53 mutation analysis

DNA was isolated from the fresh frozen tissues. DNA was used to PCR amplify a 163-bp fragment spanning the two mutational hotspots C228T and C250T in TERT promoter region; and similarly, TP53 coding regions from exons 5 to 8 were also PCR amplified. Samples were obtained as per the guidelines of Human Ethics Committee of AIIMS. PCR products were further purified and cleaned using exosap as per manufacturer's protocol. Subsequently, 2 ml of the purified amplification product was submitted to bidirectional sequencing using the BigDye Terminator Cycle Sequencing kit v1.1 (Life Technologies, Carlsbad, CA, USA). Bidirectional sequencing was performed using an ABI 3730 sequencer (Applied Biosystems) and analyzed by the Sequencing Analysis software.

The primers used:

hTERT promoter: Forward 5′-GTCCTGCCCCTTCACCTT-3′

Reverse 5′-CAGCGCTGCCTGAAACTC-3′

P53 (Exons 5–8): Forward 5′ -TGTTCACTTGTGCCCTGACT-3′

Reverse 5′- TAACCCCTCCTCCCAGAGA-3′

Forward 5′-CTTGCCACAGGTCTCCCCAA-3′

Reverse 5′-AGGGGTCAGCGGCAAGCAGA -3′

Forward 5′- TTGGGAGTAGATGGAGCCT-3′

Reverse 5′-AGGCATAACTGCACCCTTGG-3′

### Statistical analysis

In *in vitro* experiments, comparisons between groups were performed using Paired Student's *t*-test. In *in vivo* experiments, statistical analysis was done using unpaired Student's *t*-test. All values of *P* less than 0.05 were taken as significant.

## Abbreviations

hTERT: human Telomerase reverse transcriptase
ROS: reactive oxygen species
G6PD: glucose-6-phosphate dehydrogenase
TKT: transketolase
PPP: pentose phosphate pathway
GS: glycogen synthase
GBM: glioblastoma multiforme
OXPHOS: oxidative phosphorylation
R5P: ribose 5-phosphate
SOD-1: super oxide dismutase-1
DN-TERT: dominant-negative TERT
OE-Nrf2: overexpression Nrf2
E.Vector: empty vector
PAS: periodic acid Schiff
*β*-Gal: *β*-galactosidase
DHE: dihydroethidium
NAc: *N*-acetyl-l-cysteine
NAD(P)H: nicotinamide adenine dinucleotide phosphate
SFM: serum-free media
