# Supplementary material for: Nrf2-driven TERT regulates pentose phosphate pathway in glioblastoma
Source: Cell Death Dis. 2016 May 5;7(5):e2213–. doi: 10.1038/cddis.2016.117 (PMC4917655; doi:10.1038/cddis.2016.117)
Supplement: Supplementary Table 1 [file cddis2016117x1.doc]

## Supplementary Table.1

| **Genes** | **Fold Up Regulation** |
| --- | --- |
| ***ALDOB*** | **26.0642** |
| ***BPGM*** | **10.5123** |
| ***ENO3*** | **3.2132** |
| ***FBP1*** | **32.5368** |
| ***FBP2*** | **32.0888** |
| ***FH*** | **2.3198** |
| ***G6PC*** | **7.485** |
| ***GCK*** | **27.3601** |
| ***GSK3A*** | **2.0195** |
| ***GSK3B*** | **2.4691** |
| ***GYS2*** | **23.3282** |
| ***IDH3A*** | **2.5919** |
| ***PDK1*** | **2.4521** |
| ***PDK4*** | **3.191** |
| ***PGM3*** | **4.77** |
| ***RPIA*** | **5.2561** |
| ***SDHD*** | **2.7207** |
| ***UGP2*** | **2.0619** |

| **Genes** | **Fold Down Regulation** |
| --- | --- |
| ***AGL*** | **-2.6135** |
| ***ENO1*** | **-2.5955** |
| ***G6PC3*** | **-2.8799** |
| ***G6PD*** | **-3.3543** |
| ***HK2*** | **-2.5775** |
| ***IDH2*** | **-3.7477** |
| ***OGDH*** | **-2.0364** |
| ***PC*** | **-2.4385** |
| ***PGM1*** | **-2.3392** |
| ***TKT*** | **-3.8264** |
