# Supplementary figures and images for: Nrf2-driven TERT regulates pentose phosphate pathway in glioblastoma
Source: Cell Death Dis. 2016 May 5;7(5):e2213–. doi: 10.1038/cddis.2016.117 (PMC4917655; doi:10.1038/cddis.2016.117)

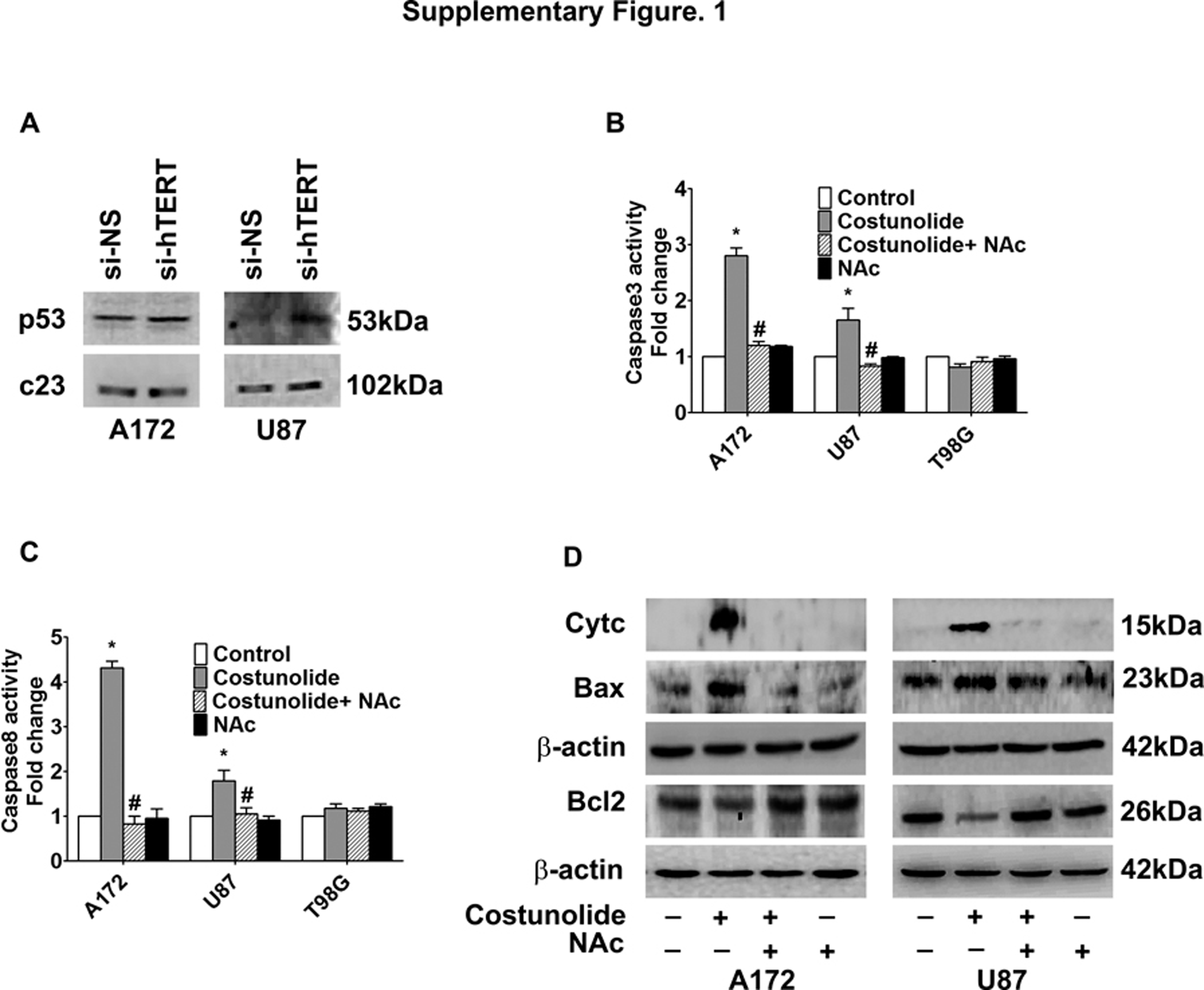

Supplement: Supplementary Figure 1 [file cddis2016117x2.tif]

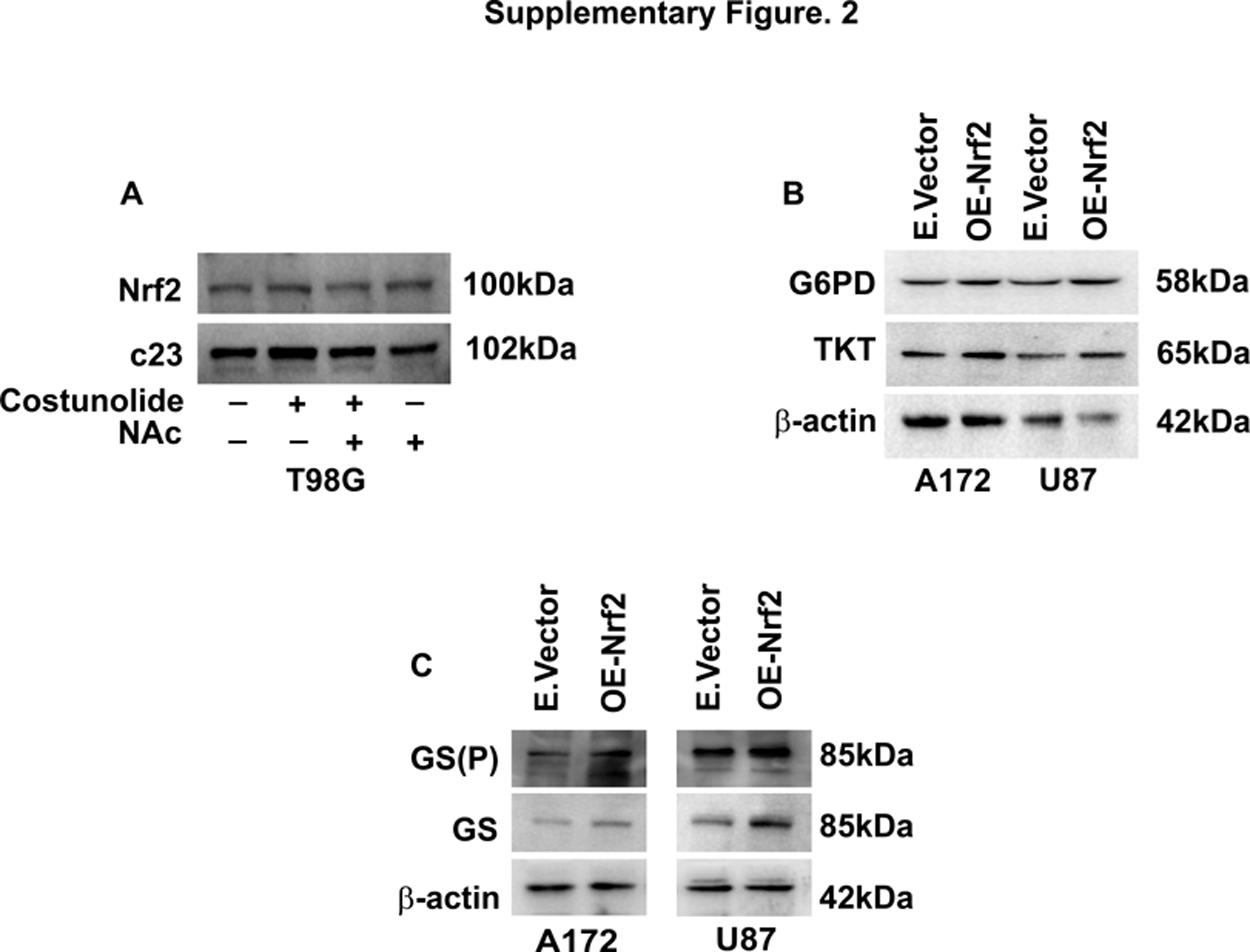

Supplement: Supplementary Figure 2 [file cddis2016117x3.tif]

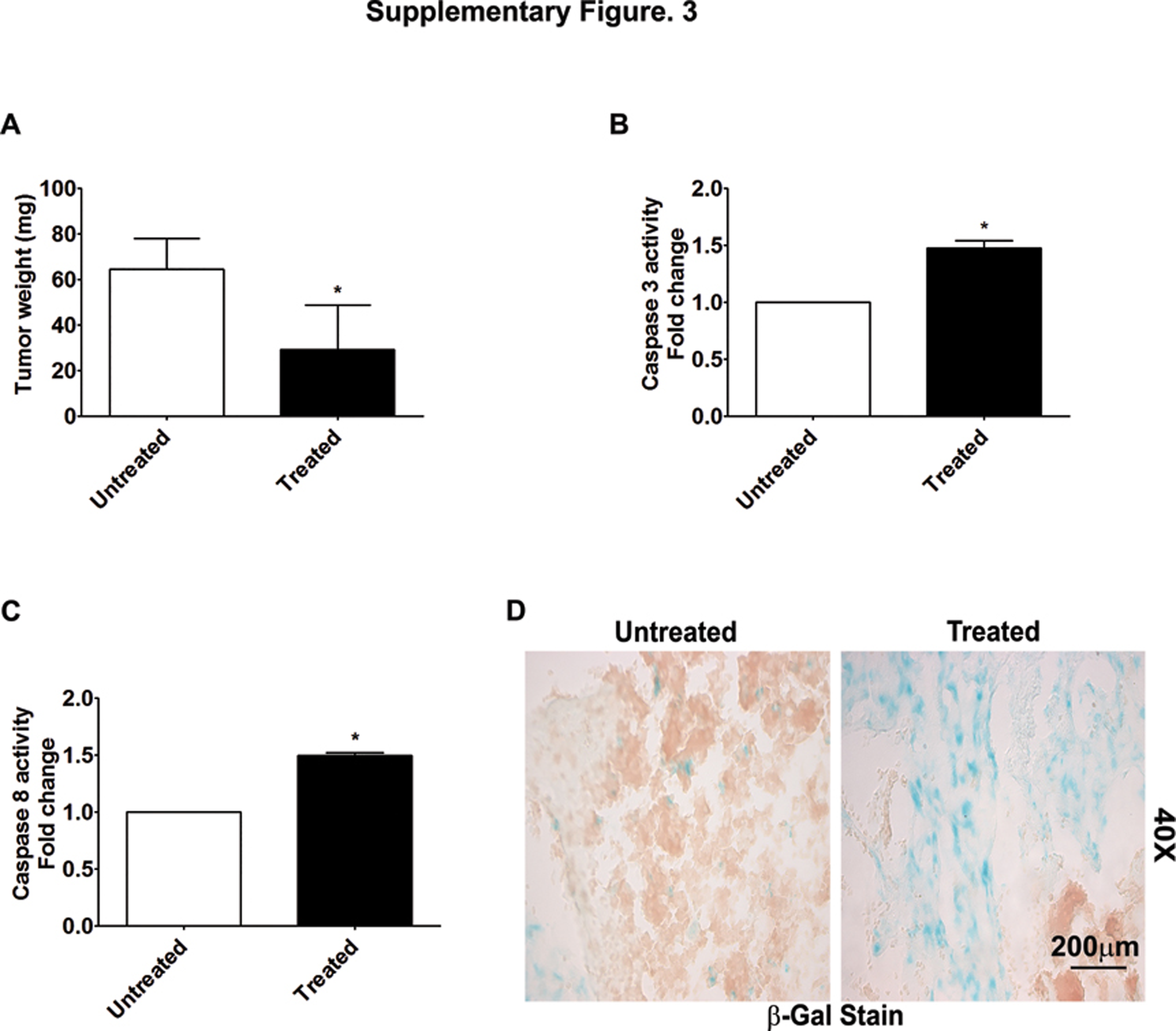

Supplement: Supplementary Figure 3 [file cddis2016117x4.tif]
