# Supplementary material for: Nrf2-driven TERT regulates pentose phosphate pathway in glioblastoma
Source: Cell Death Dis. 2016 May 5;7(5):e2213–. doi: 10.1038/cddis.2016.117 (PMC4917655; doi:10.1038/cddis.2016.117)
Supplement: Supplementary Information [file cddis2016117x5.docx]

**Supplementary Table 1.** Quantitative Real time PCR analysis demonstrating the relative transcript levels of a panel of genes associated with glucose metabolism.

Gene expression profiles of mRNA isolated from untreated and Costunolide treated U87MG glioma cells were analyzed by quantitative PCR for genes involved in glucose metabolism. Expression level of genes affected by Costunolide treatment is shown. Table represents the average data from two independent experiments.

**Supplementary Figure. 1. Costunolide induces glioma cell apoptosis.**

(A) siRNA mediated knock-down of TERT increases p53 levels as demonstrated by Western blot analysis. (B) Costunolide induces caspase 3 and (C) 8 activity in a ROS dependent manner. Value shows Caspase 3 and 8 enzyme activity in glioma cells treated with Costunolide in the presence or absence of NAc, expressed as fold change over control. (D) NAc reverses the effects of Costunolide on Bax, Bcl2 and cytochrome-c as demonstrated by Western blots analysis. Blots (A, D) are representative of three independent experiments showing similar results. Blots were re-probed for β-actin or c23 to establish equivalent loading. The values in the graph (B, C) represent the mean ± SEM from 3 independent experiments. * Significant change from control, # Significant change from Costunolide treated cells (P<0.05).

**Supplementary Figure. 2. Nrf2 affects pentose phosphate pathway and glycogen accumulation in glioma cells**. (A) Costunolide has no effect on Nrf2 level in p53 mutant T98G cells. Western blot demonstrating Nrf2 levels in T98G cells treated with different combinations of Costunolide and NAc. (B) Nrf2 over-expression increases G6PD and TKT, (C) GS(P) levels in glioma cells as demonstrated by Western blots analysis. Blots (A-C) are representative of three independent experiments showing similar results. Blots were re-probed for β-actin or c23 to establish equivalent loading.

**Supplementary Figure. 3. Costunolide inhibits tumor growth in heterotypic xenograft glioma model.** (A) Graph denotes the average tumor weights in control and treated groups (n=7). Following 20 days of Costunolide administration, the tumor mass was dissected out and weighed. Tumors from Costunolide treated group weighed significantly less as compared to the untreated controls at the end of treatment schedule. (B) Costunolide treated tumors show increased Caspase-3 and (C) Caspase-8 enzyme activity as compared to the untreated tumors. Graph depict the average enzyme activity in the tumor lysates of Costunolide treated and untreated groups (n=4). Values represented as means+/-SEM with the indicated number of animals per group. Statistical analysis was done using unpaired student’s t-test. Results were considered significant, when p value was equal to or less than 0.05. * denotes significant change from the untreated group. (D) Costunolide treated xenograft tissue sections show an increase in senescence as demonstrated by increased β-galactosidase staining.
